# pGIAK1, a Heavy Metal Resistant Plasmid from an Obligate Alkaliphilic and Halotolerant Bacterium Isolated from the Antarctic Concordia Station Confined Environment

**DOI:** 10.1371/journal.pone.0072461

**Published:** 2013-08-29

**Authors:** Suxia Guo, Jacques Mahillon

**Affiliations:** Laboratory of Food and Environmental Microbiology, Université Catholique de Louvain, Croix du Sud, Louvain-la-Neuve, Belgium; Belgian Nuclear Research Centre SCK/CEN, Belgium

## Abstract

pGIAK1 is a 38-kb plasmid originating from the obligate alkaliphilic and halotolerant *Bacillaceae* strain JMAK1. The strain was originally isolated from the confined environments of the Antarctic Concordia station. Analysis of the pGIAK1 38,362-bp sequence revealed that, in addition to its replication region, this plasmid contains the genetic determinants for cadmium and arsenic resistances, putative methyltransferase, tyrosine recombinase, spore coat protein and potassium transport protein, as well as several hypothetical proteins. Cloning the pGIAK1 *cad* operon in *Bacillus cereus* H3081.97 and its *ars* operon in *Bacillus subtilis* 1A280 conferred to these hosts cadmium and arsenic resistances, respectively, therefore confirming their *bona fide* activities. The pGIAK1 replicon region was also shown to be functional in *Bacillus thuringiensis*, *Bacillus subtilis* and *Staphylococcus aureus*, but was only stably maintained in *B. subtilis*. Finally, using an *Escherichia coli* - *B. thuringiensis* shuttle BAC vector, pGIAK1 was shown to display conjugative properties since it was able to transfer the BAC plasmid among *B. thuringiensis* strains.

## Introduction

Organisms with optimal pH for growth in excess of pH 9 are defined as alkaliphiles [1]. Alkaliphilic bacteria can be further divided into two groups: facultative and obligate alkaliphiles. The bacteria from the former group can grow well below or at pH 8, while the latter group cannot grow well below or at pH 8 [2]. Since the first report of alkaliphilic Bacilli (*Bacillus alcalophilus*) by Vedder in 1934 [3], numerous alkaliphiles have been discovered, not only under extreme alkaline conditions, such as soda lakes [4], [5], [6], [7], [8], but also in more conventional environments such as gardens and soil [9], [10], [11]. Alkaliphilic bacteria are also relatively frequent in confined environments [12]. Due to their ability of producing various types of valuable enzymes, such as alkaline proteases, alkaline amylases or alkaline cellulases, these bacteria have been widely investigated for their potential uses in biotechnological applications [1], [13].

Although little is known about extrachromosomal elements from alkaliphilic bacteria, plasmid profiles from several alkaliphilic bacteria have been reported to harbor both small (<20 kb) and large (>20 kb) plasmids [14], [15]. Similarly, the development of genomic sequencing has revealed the presence of plasmids in several alkaliphic bacteria [16], [17], [18], [19], [20], [21], [22] although their actual functions have so far remained unclear. One notable exception is the presence of the cadmium resistance determinants found on both pBpOF4-01 and pBpOF4-02 plasmids of the alkaliphilic *Bacillus pseudofirmus* OF4 [20].

Unlike open environments, confined settings can influence the microbial prevelance and diversity [23]. The issue of confined environments not only involves bacterial contamination [24], but also horizontal gene transfers mediated by mobile genetic elements (MGEs) [25] which can indeed mediate the exchange of genetic determinants for antibiotic or heavy metal resistances, or even virulence, among bacterial populations.

During our study on the microflora present in the confined Concordia station in Antarctica, an obligately alkaliphilic and halotolerant bacterium named JMAK1 was isolated. The earlier study showed that this strain was resistant to arsenate (>40 mM) at pH 9.0. In the course of the characterization of this alkaliphilic bacterium, we discovered that its genome contained at least two large plasmids (>20 kb). In order to get more insights into the potential biological role(s) of these elements, the nucleotide sequence of the smallest detectable plasmid (38 kb) was determined and the putative functions of some of its genetic determinants explored using heterologous mesophilic and neutrophilic bacterial hosts.

## Materials and Methods

### Bacterial Strains and Plasmids

All the bacterial strains and plasmids used in this study are listed in Table 1. The JMAK1 strain was originally isolated from a stairway step inside the Antarctic Concordia station by the method previously described by Van Houdt *et al*. (2009) [23], except that alkaline medium was used to cultivate the bacteria. It was routinely incubated in pH 9.0 TY medium (10 g tryptone, 5 g yeast extract, 5 g NaCl, 1 g K_2_HPO_4_, 0.02 g MgSO_4_ per liter) buffered with 100 mM NaHCO_3_/Na_2_CO_3_ to a final pH 9 at 30°C. This bacterium was kept in the Deutsche Sammlung von Mikroorganismen und Zellkulturen (DSMZ) and Belgian Co-ordinated Collections of Micro-organisms (BCCM) under numbers DSM 24526 and LMG 27139, respectively. It is currently under characterization.

**Table 1:** Strains and plasmids used in this study.

Strains	Main characteristics	Reference/Source
JMAK1	Alkaliphilic, Bacillaceae	This study
GBJ001	Sm^R^ mutant of *B. thuringiensis* serovar *israelensis* 4Q7, plasmid free	[30]
GBJ002	Nal^R^ mutant of *B. thuringiensis* serovar *israelensis* 4Q7, plasmid free	[31]
BMB171	Mutant of *B. thuringiensis* serovar *kurstaki* YBT-1463	[26]
BEST2125	Tet^R^ mutant of *B. subtilis* 168	[27]
NCTC 8325 cA2	Mutant of *S. aureus* NCTC8325, plasmid free	Lab stock
H3081.97	*B. cereus*, wild type, Cd^S^	[28]
DH10B	*E. coli*	Lab stock
1A280	Mutant of *B. subtilis* 168, As^S^	[29], BGSC*
**Plasmids**		
pIndigoBAC536	BAC vector	[32]
pEMB0557	Shuttle BAC vector	[33]
pDG780	Cloning vector	[35]
pDG646	Cloning vector	[35]
pHT304	Cloning vector	[34]
pGIAK1	38,362-bp, Cd^R^ and As^R^ plasmid	This study
pGIAK11	pIndigoBAC536 vector carrying the entire pGIAK1 plasmid, Cm^R^	This study
pGIAK12	pEMB0557 vector carrying the entire pGIAK1 plasmid, Cm^R^, Ery^R^	This study
pGIAK13	pDG780 vector carrying the 7.0-kb *Eco*RI fragment of pGIAK1, Kan^R^, Amp^R^	This study
pGIAK13A	pDG646 vector carrying the 5.5-kb *Eco*RI-*Sca*I fragment of pGIAK1, Ery^R^, Amp^R^	This study
pGIAK14	pHT304 vector carrying a 3.8-kb *Bam*HI-*Pvu*II fragment of pGIAK1, Cd^R^, Ery^R^, Amp^R^	This study
pGIAK15	pHT304 vector carrying the 11.3-kb *Bam*HI-*Pst*I fragment of pGIAK1, As^R^, Ery^R^, Amp^R^	This study

R resistant; ^S^sensitive;

* Bacillus Genetic Stock Center.

*Bacillus thuringiensis* BMB171 [26], *Bacillus subtilis* BEST2125 [27] and *Staphylococcus aureus* NCTC8325 cA2 were used as hosts for testing the replication of pGIAK1, while *Bacillus cereus* H3081.97 [28] and *B. subtilis* 1A280 [29] were used as cadmium and arsenic sensitive host, respectively. The *Escherichia coli* mutant strain DH10B was used as cloning host while *B. thuringiensis* GBJ001 (Sm^R^) [30] and GBJ002 (Nal^R^) [31] were used as host strains in the mating experiments.

Lysogeny broth (LB) medium was used for all neutrophilic bacterial growth unless otherwise indicated. For solid medium, 15 g agar was added in 1 L medium. *B. thuringiensis*, *B. cereus* and *B. subtilis* were grown at 30°C, whereas *E. coli* and *S. aureus* were incubated at 37°C. When required, antibiotics (Sigma-Aldrich, St. Louis, MO) were added to the media in final concentrations (µg/mL) of: chloramphenicol (Cm), 12.5; ampicillin (Amp), 100; erythromycin (Ery), 5 for *B. subtilis* and 25 for *B. thuringiensis* and *B. cereus*; streptomycin (Sm), 100; nalidixic acid (Nal) 15; and kanamycin (Kan), 50.

The bacterial artificial chromosome (BAC) vectors pIndigoBAC536 [32] and pEMB0557 [33] were used to clone the complete plasmid of pGIAK1, the cloning vector pHT304 [34] was used for sub-cloning library construction, and the pDG780 and pDG646 vectors [35] were used for replicon cloning. Both of them contain a Gram-negative replicon, a *bla* marker (active in *E. coli*) and antibiotics markers (for selection in *Bacillus* spp.).

### Plasmid Constructs and DNA Manipulation

Plasmids from the JMAK1 and *Bacillus* strains were isolated following the procedure of Andrup *et al.* [36]. Plasmid sizes were estimated by comparing with the reference plasmids of *B. thuringiensis* serovar *israelensis* AND508 [36] **.** Plasmid preparation from *E. coli* and electroporation, DNA restriction and ligation were performed according to Sambrook and Russell [37]. DNA transformation was carried out through electroporation in a Gene Pulser Xcell apparatus (Bio-Rad Laboratories, Hercules, CA). The electroporation of *B. thuringiensis* or *B. cereus* [38], *B. subtilis* [39] and *S. aureus* [40] were performed as previously described. All the enzymes used in this study were purchased from Promega (Promega Corp., Madison, WI).

Plasmid pGIAK1 was digested with *Bam*HI and ligated to the *Bam*HI digested pIndigoBAC536 and pEMB0557 vectors, generating pGIAK11 and pGIAK12, respectively. Then pGIAK11 was cleaved by *Hin*dIII, *Eco*RI, *Pvu*II and *Pst*I separately and ligated to pHT304, cleaved with the *Hin*dIII, *Eco*RI, *Sma*I and *Pst*I correspondingly, giving rise to four sub-cloning libraries. Plasmid pGIAK12 was introduced by electroporation into *B. thuringiensis* GBJ002, and the resulting GBJ002 (pGIAK12) was used as donor in the mating experiments.

The pGIAK1 replicon cloning and stability tests were performed following the method described by Huang *et al*. [41]. Total plasmid DNA from the strain JMAK1 was digested with *Eco*RI and ligated to *Eco*RI-digested pDG780 vector. The resulting recombinant plasmids were electroporated into *B. thuringiensis* BMB171 and selected on kanamycin plates. The Kan^R^ transformants were transferred into *E. coli* DH10B and recombinant plasmid containing the pGIAK1 replicon was confirmed by end-sequencing using the primers 780-RP (5′-TGCCTCCTCATCCTCTTCAT-3′) and M13-FP (5′-TGTAAAACGACGGCCAGT-3′).

### Plasmid pGIAK1 Nucleotide Sequencing and Analysis

Plasmids from the sub-cloning libraries of plasmid pGIAK1 were extracted and sequenced by ABI 3730xl DNA Sequencer at the GATC Biotech Company (GATC Biotech, Konstanz, Germany). Sequence data were assembled and analyzed using the SeqMan 5.00 program from the DNAStar software package (Lasergene Inc., Madison, Wis.). Finally the sequence gaps were filled by PCR amplification using primers targeting the end of neighboring contigs and then sequenced. The complete sequence was annotated using the ISGA pipeline [42]. Each predicted protein sequence was manually analyzed using the BLASTP program against the GenBank database [43]. The minimal length of overlap is 80%. The genetic map of plasmid pGIAK1 was visualized in CGView program [44]. The cloning scheme was generated using Clone Manager 8.0 (Scientific and Educational Software, Cary, NC).

### Nucleotide Sequence Accession Numbers

The 16S rRNA gene sequence of strain JMAK1 and the complete nucleotide sequence of plasmid pGIAK1 have been submitted to the GenBank database under accession numbers of JF729319 and JX406743, respectively.

### Cadmium and Arsenic Resistance Assays

To determine the heavy metal resistances of pGIAK1, the recombinant plasmid pGIAK14 containing *cad* operon from the sub-cloning library was selected by PCR using primers Cad-F (5′-GCTTAGCTGTGCTTGTCGTG-3′) and Cad-R (5′-ATCCGTTACACCGACATGGT-3′). The recombinant plasmid pGIAK15 containing the *ars* operon was screened by PCR with primers ArsB-F (5′-TCCACCAGGAGATTTTCACA-3′) and ArsB-R (5′-GGAAATGCCGTGCTTATGTT-3′). Plasmids pGIAK14 and pGIAK15 were transferred into strain *B. cereus* H3081.97 and *B. subtilis* 1A280, respectively, via electroporation. These two recombinants were used in the cadmium and arsenic resistance tests, respectively.

Cadmium chloride (CdCl_2_) and sodium arsenate dibasic heptahydrate (Na_2_HAsO_4_•7H_2_O) were prepared as 0.1 M and 0.5 M stock solutions, respectively. The cadmium resistance was tested in LB medium while the arsenate resistance assay was performed in low-phosphate medium (LPM) supplemented with 1% tryptone, as described by Branco *et al.* [45]. This medium contains (in g/L), Tris 6.06 (pH 7.0), NaCl 4.68, KCl 1.49, NH_4_Cl 1.07, Na_2_SO_4_ 0.43, MgCl_2_.6H_2_O 0.2, CaCl_2_.2H_2_O 0.03, Na_2_HPO_4_.12H_2_O 0.23, glucose 5.0 and tryptone 10. All the assays were carried out in 96-well micro-plates containing different concentrations of heavy metals. Each well contained 200 µL of media and 10 µL of overnight bacterial culture. Bacterial growth was measured by optical density at 595 nm on a Multiskan FC Microplate Photometer (Thermo Fisher Scientific, Inc., Waltham, MA) after 20 h of growth at 30°C.

### Mating Tests

Conjugation assays were performed using the drop-on-drop method [46] with some modifications. 10 µL of overnight cultures of donor and recipient were mixed on LB agar plate and incubated for 24 h at 30°C. The lawn cultures were re-suspended in 4 mL LB medium, and 100 µL of the suspension was diluted and plated on LB agar plates in five replicates, containing antibiotics for recipients, and incubated at 30°C for 12 h. The remaining suspension was plated on LB agar plates containing corresponding antibiotics to select the transconjugants. The transconjugants were confirmed by PCR. Spontaneous mutants were discriminated from transconjugants by checking for their antibiotic resistances.

## Results and Discussion

### Bacterial Strain JMAK1

**Figure 1:**
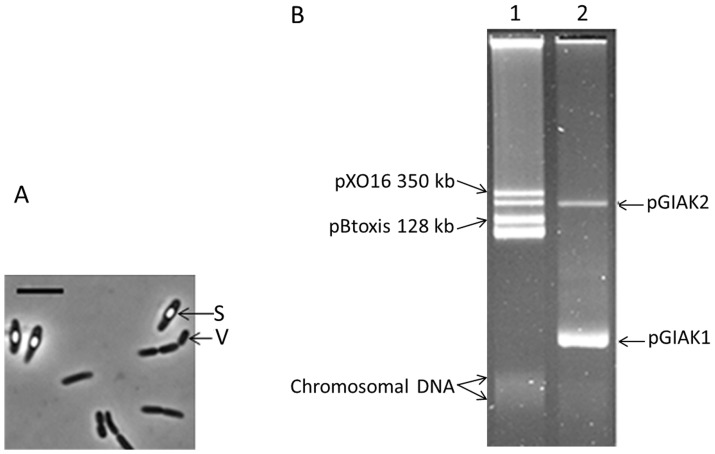
Cells and plasmids of strain JMAK1. (A) Bacterial cells were observed under a BX50 phase contrast microscope (Olympus, Japan). V, vegetative cell; S, spore. The bar is 5 µm. (B) Plasmid profile of strain JMAK1. The plasmid preparation was run in a 0.5% agarose gel and stained with Ethidium Bromide. The chromosomal DNA referred to the degraded linear chromosomal material. Lane 1, *B. thuringiensis* serovar *israelensis* AND508; Lane 2, JMAK1.

JMAK1 is an obligate alkaliphilic, aerobic and halotolerant spore-forming bacterium. The vegetative cells are rod-shaped (Figure 1A). The bacterium cannot grow at pH 7.0 or lower and displayed an optimal growth between pH 8.5 to pH 9.5. Although the bacterium sustained NaCl concentration up to 17% (w/v), its optimum concentration was between 1 - 3% (w/v) at pH 9. The 16S rRNA gene sequence of JMAK1 displayed 99.5% identity to that of the alkaliphilic and halotolerant “Bacillales bacterium” CPCC100153 [47] (GenBank accession No. HQ179102) isolated from alkaline soil of Hoh Xil (Qinghai-Tibet plateau). However, this strain has not been taxonomically validated yet. The closest validly described strain was *Geomicrobium halophilum* BH1^T^ whose 16S rRNA shared only 93.2% identity.

### pGIAK1 main Features

Analysis of the plasmid content of JMAK1 (Figure 1B) revealed the presence of at least two large molecules named pGIAK1 and pGIAK2. Based on the reference plasmids of *B. thuringiensis* serovar *israelensis* AND508, their sizes were estimated to be ca. 40 and 200 kb, respectively. As shown in Figure 1B, the relative DNA intensity of pGIAK1 was much stronger than that of its large co-resident pGIAK2, suggesting a higher copy number for pGIAK1. No smaller plasmid (<20 kb) was detected in this strain.

**Figure 2:**
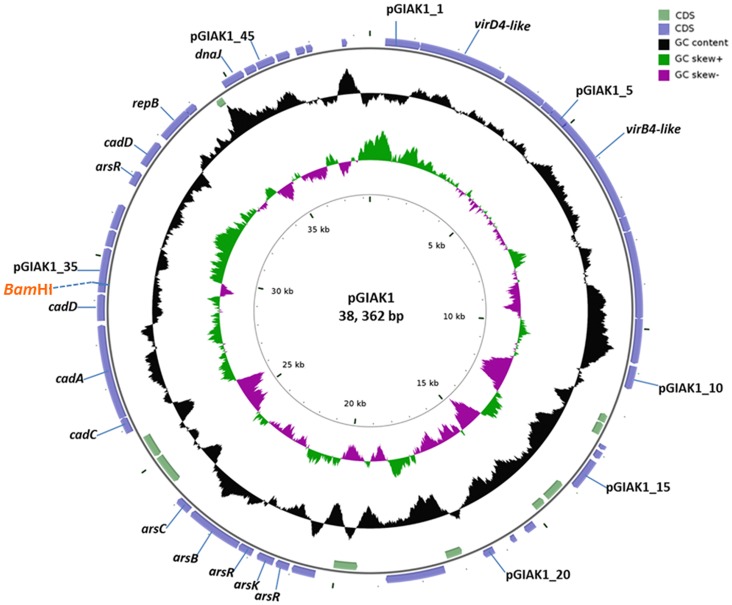
Physical and genetic map of plasmid pGIAK1. The map was visualized in CGView [44]. The outer circles indicate the predicted CDS. The blue and light green block arrows show the predicted genes with different transcription directions. The numbers beside the arrows indicate the corresponding genes. The black circle represents the GC content and the green/purple circles represent the GC-skew. The predicted genes involved in replication, cadmium resistance, arsenic resistance and conjugation are indicated. The single cloning site *Bam*HI is also indicated.

**Figure 3:**
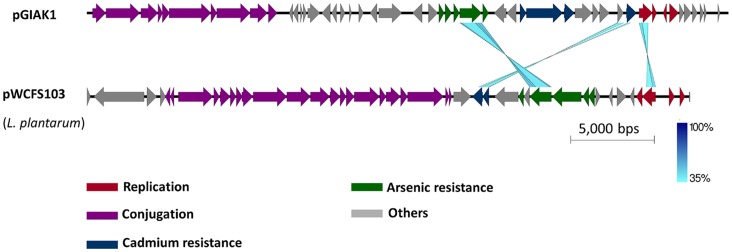
Comparison of pGIAK1 and the heavy metal resistant plasmid pWCFS103 from *L. plantarum*. The displayed bar represents a length of 5,000 basepairs. The conserved regions are blue-shaded, the color intensity indicating the identity levels (from 35 to 100%). Genes that displayed homologies are indicated by the colored boxes shown below. The alignment was made by the Easyfig 2.1 program [49] **.**

The complete sequence of pGIAK1 was determined to be 38,362 bp, with a G+C content of 37.5%. By BLASTN [48] analysis, 95% of the plasmid DNA sequence had no homologue in the GenBank database. However, it potentially contained 49 ORFs, from which 40 had BLASTP significant similarities to sequences in the GenBank protein database (see Table S1). Based on these homologies, a potential replication region, putative arsenic and cadmium resistance operons, and potential conjugation-related genes could be identified (Figure 2, see below). Using the Easyfig 2.1 software [49], functional similarities could be identified between pGIAK1 and the heavy metal resistant plasmid pWCFS103 [50] from the distantly related *Lactobacillus plantarum*. As shown in Figure 3, both plasmids contain genes related to conjugation, cadmium and arsenic resistance, although the genes are arranged differently. Their conjugation regions shared no similarities while their ArsB, CadD and RepB proteins shared 68%, 36% and 38% identity, respectively.

### pGIAK1 Replication Region

As indicated in Table S1, gene pGIAK1_40 encodes a putative 30 kDa ParA nucleotide binding domain protein. ParA is a membrane-associated ATPase involved in the segregation of plasmid and bacterial chromosome [51]. The predicted amino acid sequence is quite similar to the plasmid copy control protein RepB of *Enterococcus faecalis* plasmids pEJ97 (46.7% identity), pAD1 (40.1% identity) and pAM373 (39.2% identity) [52], [53], [54], and as well as *L. plantarum* plasmid pWCFS103 (38% identity) mentioned above. A small hypothetical gene pGIAK1_41 encoding a 9.9 kDa polypeptide partly overlaps with pGIAK1_40. A similar structure was also found downstream of the *repB* genes in *L. plantarum* plasmid pWCFS103 (Figure 3) [50], *E. faecalis* pAD1 [52]. Following these two genes, pGIAK1_43 encodes a 21-kDa DnaJ domain protein. A *dnaJ-like* gene was also found downstream of *repB* gene on the *E. faecalis* conjugative plasmid pRE2*5* [55]. The two *DnaJ* proteins show 37.3% identity. Protein DnaJ was first known to stimulate the ATPase activity of DnaK, the bacterial Hsp70 homolog, and to help replicate phage DNA in host cells [56], [57], [58]. The genes from pGIAK_44 to pGIAK_49 are all hypothetical.

A 7.0-kb *Eco*RI fragment (see Figure S1A) encompassing the potential replication region of pGIAK1 (from pGIAK1_38 to pGIAK1_49, Table S1) was cloned into the *E. coli* pDG780 vector [35], giving rise to pGIAK13. This recombinant plasmid was electroporated and shown to replicate in *B. thuringiensis* BMB171. A similar result was obtained by cloning the pGIAK1 segment between *Sca*I and *Eco*RI (see Figure S1A) into pDG646, giving rise to pGIAK13A. The recombinant plasmid pGIAK13 was further electroporated into *B. subtilis* BEST2125 and *S. aureus* NCTC 8325 cA2. Transformants were obtained in both bacterial hosts. This result indicated that the pGIAK1 replicon is able to replicate in at least two *Bacillus* and one *Staphylococcus* species.

When the pGIAK13-bearing hosts were cultured without antibiotics for about 40 generations at 30°C, the recombinant plasmid was unstable in *B. thuringiensis* BMB171 (2% stability) and *S. aureus* NCTC 8325 cA2 (<0.1%), but highly stable in *B. subtilis* BEST2125 (100% stability). Interestingly, pGIAK1 was also found to be stably maintained in its original host since no cured strain could be obtained, even after several repeated trials (data not shown).

### pGIAK1 Cadmium Resistance

pGIAK1 carries two copies of *cadD*-like genes (pGIAK1_34 and 39, Figure 2) whose corresponding putative proteins shared 69% identities. Gene pGIAK1_39 is close to the replication region while pGIAK1_34 is located downstream of an intact potential cadmium operon encoding the cadmium efflux system accessory protein CadC and the cadmium-translocating P-type ATPase CadA. In *S. aureus*, a cadmium resistance operon on plasmid pI258 contains *cadA* and *cadC* genes [59]. The 724-aa CadA protein was reported to function as an energy-dependent cadmium efflux ATPase, while the 122-aa CadC protein served as a transcription regulator of the *cad* operon. It was also shown that the expression of *cadD* in *S. aureus* and *B. subtilis* resulted in low-level resistance to cadmium [60].

**Figure 4:**
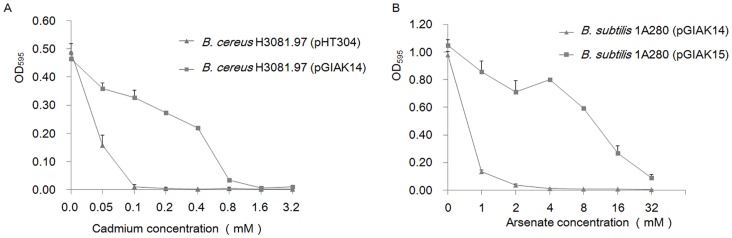
Cadmium and arsenate resistance assays. Cadmium resistance assay (A) was performed in LB medium and arsenate resistance assay (B) was performed in low phosphate medium containing increasing concentrations of Cd^2+^ (A) or As^5+^ (B). Bacterial growth was measured by optical density at 595 nm after 20 h of growth at 30°C. Each data point represents the mean value of triplicate cultures. The error bars indicate the standard deviation values. OD_595_, optical density at 595 nm.

In order to test whether the cadmium operon from pGIAK1 could confer cadmium resistance to *Bacillus,* the recombinant plasmid pGIAK14 containing the *cadA*, *cadC* and *cadD* genes (*Pvu*II to *Bam*HI region, see Figure S1B), cloned into pHT304 was introduced into the cadmium sensitive strain *B. cereus* H3081.97 generating recombinant *B. cereus* H3081.97 (pGIAK14). As shown in Figure 4A, *B*. *cereus* H3081.97 containing the vector pHT304 could not grow in the medium with cadmium chloride at a concentration higher than 0.1 mM, while *B. cereus* H3081.97 (pGIAK14) was able to grow in the presence of cadmium chloride up to 0.8 mM (Figure 4A). This result indicated that the cadmium resistant determinant from the alkaliphilic bacterium JMAK1 is indeed functional, as demonstrated by its activity in the heterologous and mesophilic host *B. cereus* H3081.97.

### pGIAK1 Arsenic Resistance

Plasmid pGIAK1 also potentially encodes a set of genes that may be involved in arsenic resistance, including *arsR*, *arsK*, *arsB* and *arsC* (pGIAK1_25 to pGIAK1_29, Figure 2). In bacterial *ars* operons, *arsR* encodes the repressor of arsenic resistance, *arsB* encodes a membrane transport protein that catalyzes extrusion from the cells, and *arsC* encodes an arsenate reductase that reduces arsenate to arsenite, which is the transport substrate [61], [62], [63]. The function of ArsK (former YqcK) is not well characterized yet, although it was shown that its deletion in *B. subtilis* affects aerobic growth and sporulation in the presence of arsenic [64]. The structure of the pGIAK1 *ars* operon is also similar to those of the *Staphylococcus* pI258 plasmid [65] and the *B. subtilis skin* element (*skin*, for sigK intervening) [66] except that pGIAK1 bears two copies of the *arsR* regulatory gene and one copy of *arsK*.

To verify the activity of this *ars* operon, a recombinant plasmid pGIAK15 containing the pGIAK1 11.3-kb *Bam*HI*-Pst*I fragment was electroporated into the arsenic sensitive strain *B. subtilis* 1A280. This fragment covered the *Bam*HI to *Ps*tI pGIAK1 region (see Figure S1B). As illustrated in Figure 4B, the recombinant *B. subtilis* 1A280 (pGIAK15) was more than 20 times more resistant to arsenate than the negative control *B. subtilis* 1A280 (pGIAK14). When *B. subtilis* 1A280 (pGIAK15) was exposed to arsenite, it showed similar resistance (data now shown). These results demonstrated that the *ars* operon from plasmid pGIAK1 endows arsenic resistance to *B. subtilis* 1A280.

### pGIAK1 Transfer Activity

The plasmid pGIAK1 encodes several polypeptides (ORFs 1 to 10) that have conserved domains of conjugation related elements. As shown in Table S1, pGIAK1_1 encodes a Replic_Relax family protein. Proteins from this family are essential for plasmid replication and plasmid DNA relaxation [67], [68]. The predicted protein of pGIAK1_2 has VirD4 conserved domain at its NH2-terminus, while product of pGIAK1_6 has a AAA-like domain which is similar to VirB4 family protein (Figure 2). In *Agrobacterium tumefaciens*, VirD4 is known to function as a coupling protein while VirB4 protein is postulated to energize the substrate export by ATP-driven conformational changes during the process of plasmid conjugation [69]. Both of them are essential elements of the VirB/VirD4 type IV secretion system which can mediate plasmid DNA mobilization [70]. Gene pGIAK1_7 encode a putative lipoprotein. In the case of pheromone-inducible conjugative plasmid pAD1 of *E. faecalis*, the pheromone sequences appeared within the carboxyl termini of signal sequences of lipoprotein precursors [71]. Moreover, pGIAK1_8 encodes a putative membrane protein and pGIAK1_9 encodes a potential cell wall hydrolase protein.

Taken together, these features suggest that pGIAK1 is a conjugative plasmid. In order to verify this assumption, the recombinant plasmid pGIAK12 containing the entire pGIAK1 cloned into the pEMB0557 vector was transferred into *B. thuringiensis* GBJ002 (Nal^R^) by electroporation. Mating experiments were then performed between GBJ002 (pGIAK12) (Ery^R^ and Nal^R^, donor) and *B. thuringiensis* GBJ001 (Sm^R^, recipient). Erythromycin and streptomycin resistant transconjugants were obtained at frequencies from 10^−9^ to 10^−8^ transconjugants per recipient. No mobility was observed when mating GBJ002 (pEMB0557) (Ery^R^ and Nal^R^, donor) with GBJ001 (Sm^R^, recipient). This result showed that pGIAK1 is likely a conjugative plasmid. Its low transfer efficiencies could be due to a pheromone-inducible conjugative phenomenon. The conjugative mechanism still needs further investigation.

### pGIAK1 Cryptic Features

In addition to the replicon, conjugation-related elements and heavy metal resistant determinants, plasmid pGIAK1 encodes other putative proteins that may have important effects on the biology of the host organism (Table S1). For instance, gene pGIAK1_15 encodes a putative methyltransferase protein while pGIAK1_23 encodes a potential tyrosine recombinase related to a *B. subtilis* phage integrase. The predicted product of gene pGIAK1_16 contains spore coat protein X and V domain and could therefore be involved in the sporulation of strain JMAK1. Finally, a potential potassium transport protein is encoded by pGIAK1_35. The function and role of these genes remain to be confirmed.

## Conclusions

In this study, we cloned, sequenced, and analyzed the function of a 38-kb plasmid from an alkaliphilic and halotolerant bacterium JMAK1 isolated from the confined environment of the Antarctic Concordia station. Based on *rrnB* 16S ribosomal RNA gene sequence alignment this strain appears to be most closely related to another new “Bacillales bacterium” CPCC100153 [47] isolated from the Qinghai-Tibet plateau.

The arsenic resistance of the strain JMAK1 prompted us to further investigate the genetic determinants of this metalloid resistance from a new alkaliphilic isolate. Interestingly, plasmid sequencing showed that the plasmid pGIAK1 harbors not only arsenic but also cadmium resistance genes. Both heavy metal and metalloid resistance were confirmed by cloning their corresponding genetic determinants in sensitive *Bacillus* strains. Interestingly, the *cad* genetic determinants include three loci: one putative *cadA/cadC* operon and two *cadD* genes. The host strain was originally isolated from a stair step inside the Concordia station. The real functions of these modules still need to be determined for this novel and intriguing alkaliphilic bacterium whose genome sequencing is in progress.

pGIAK1 replicon is functional in *B. thuringiensis*, *B*. *subtilis*, and *S. aureus* and could therefore be used as shuttle cloning vector between *E. coli*, *Bacillus* spp., *Staphylococcus* spp. and alkaliphilic Bacillaceae strains. Moreover, pGIAK1 is also self-mobilizable and could therefore serve as appropriate tool for further investigations on plasmid exchanges among alkaliphiles or between alkaliphiles and neutrophilic Gram-positive bacteria.

## Supporting Information

Figure S1 **The cloning scheme of recombinant plasmids pGIAK13, pGIAK13A, pGIAK14 and pGIAK15.** (A) pGIAK13 and pGIAK13A; (B) pGIAK14 and pGIAK15. The restriction enzyme sites used for each cloning are indicated. The predicted genes of plasmid pGIAK1 with different transcription directions are presented in blue and light green block arrows. The numbers beside the arrows correspond to the predicted genes encoded by pGIAK1.
(TIF)

Table S1 **Predicted genes in pGIAK1.**
(DOCX)
