# Supplementary figures and images for: pGIAK1, a Heavy Metal Resistant Plasmid from an Obligate Alkaliphilic and Halotolerant Bacterium Isolated from the Antarctic Concordia Station Confined Environment
Source: PLoS One. 2013 Aug 29;8(8):e72461. doi: 10.1371/journal.pone.0072461 (PMC3756968; doi:10.1371/journal.pone.0072461)

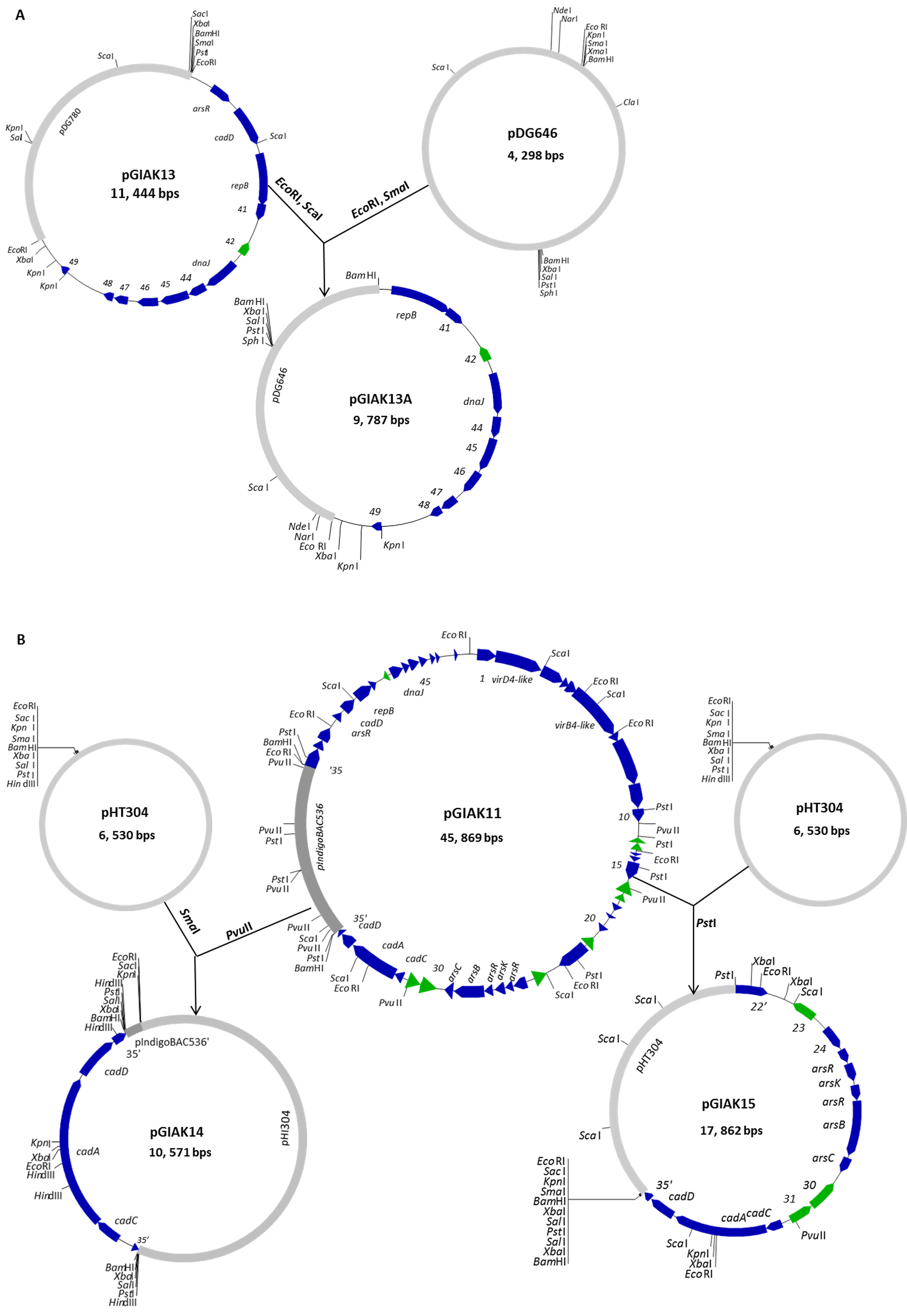

Supplement: Figure S1 — The cloning scheme of recombinant plasmids pGIAK13, pGIAK13A, pGIAK14 and pGIAK15. (A) pGIAK13 and pGIAK13A; (B) pGIAK14 and pGIAK15. The restriction enzyme sites used for each cloning are indicated. The predicted genes of plasmid pGIAK1 with different transcription directions are presented in blue and light green block arrows. The numbers beside the arrows correspond to the predicted genes encoded by pGIAK1. (TIF) [file pone.0072461.s001.tif]
