# Supplementary material for: pGIAK1, a Heavy Metal Resistant Plasmid from an Obligate Alkaliphilic and Halotolerant Bacterium Isolated from the Antarctic Concordia Station Confined Environment
Source: PLoS One. 2013 Aug 29;8(8):e72461. doi: 10.1371/journal.pone.0072461 (PMC3756968; doi:10.1371/journal.pone.0072461)
Supplement: Table S1 — Predicted genes in pGIAK1. (DOCX) [file pone.0072461.s002.docx]

**Table S1. Predicted genes in pGIAK1**

| **Name** | **CDS** | **# residues** | **Predicted function** | **Top hits in GenBank database^*^(accession number) (identity in overlapping segment) (E-value)** |
| --- | --- | --- | --- | --- |
| **pGIAK1_1** | 346-1149 | 267 | Putative DNA relaxation protein | *Bacillus pseudofirmus* OF4 hypothetical protein BpOF4_21889 (ADC52373) (35% in 226 aa) (1e-28)  Contains Replic_Relax conserved domain |
| **pGIAK1_2** | 1151-3181 | 676 | Putative VirD4 family protein | *Bacillus cereus* H3081.97 hypothetical protein (ACI30543) (32% in 727 aa) (1e-117)  Contains VirD4 conserved domain |
| **pGIAK**1_3 | 3243-4262 | 339 | Putative conjugative transposon-related protein | *B. cereus* VD045 hypothetical protein IIE_06286 (EJR25249) (31% in 344 aa) (2e-45)  Contains TcpC conserved domain |
| **pGIAK1_4** | 4263-4538 | 91 | Hypothetical protein | *Staphylococcus pseudintermedius* membrane protein (WP_020219837) (25% in 87 aa) (0.029) |
| **pGIAK1_5** | 4522-4965 | 147 | Putative conjugative transposon membrane protein | *B. cereus* VD045 hypothetical protein IIE_06284 (EJR25247) (41% in 145 aa) (4e-30)  Contains TcpE conserved domain |
| **pGIAK1_6** | 4970-7432 | 820 | Putative VirB4 family protein | *B. cereus* VD045 hypothetical protein IIE_06283 (JR25246) (59% in 817 aa) (< 5e-179)  Contains AAA-like conserved domain |
| **pGIAK1_7** | 7429-7773 | 114 | Putative lipoprotein | *Staphylococcus warneri* L37603 hypothetical protein STAWA0001_1742 (EEQ80792) (27% in 117 aa) (3e-05) |
| **pGIAK1_8** | 7779-9776 | 665 | Putative membrane protein | *B. cereus* VD045 hypothetical protein IIE_06281 (EJR25244) (34% in 563 aa) (7e-103) |
| **pGIAK1_9** | 9773-10804 | 343 | Cell wall hydrolase family protein | *Oceanobacillus kimchii* hypothetical protein (WP_017795730) (54% in 335 aa) (3e-121)  Contains Peptidase_M23 conserved domain |
| **pGIAK1_10** | 10858-11397 | 179 | Hypothetical protein | *O. kimchii* hypothetical protein (WP_017795731) (35% in 179 aa) (4e-25) |
| **pGIAK1_11** | 12318-12109 | 69 | Hypothetical protein | *Bacillus clausii* KSM-K16 hypothetical protein ABC0518 (YP_174019) (56% in 66 aa) (8e-15) |
| **pGIAK1_12** | 12632-12333 | 99 | Hypothetical protein | *Lentibacillus* sp. Grbi hypothetical protein (WP_010529275) (49% in 85 aa) (4e-15)  Contains DUF2642 conserved domain |
| **pGIAK1_13** | 12779-12895 | 38 | Hypothetical protein | No hits |
| **pGIAK1_14** | 12940-13128 | 62 | Hypothetical protein | No hits |
| **pGIAK1_15** | 13205-13945 | 246 | Methyltransferase domain protein | *Desmospora* sp. 8437 type 12 methyltransferase (EGK13388) (44% in 236 aa) (5e-79)  Contains Methyltransferase conserved domain |
| **pGIAK1_16** | 14561-14049 | 170 | Spore coat protein X and V domain protein | *Bacillus cellulosilyticus* DSM 2522 spore coat protein X/V (ADU28581) (59% in 142 aa) (1e-35)  Contains Spore Coat Protein X and V domain |
| **pGIAK1_17** | 14931-14650 | 93 | Hypothetical protein | No hits |
| **pGIAK1_18** | 15176-15436 | 86 | Hypothetical protein | *Planococcus halocryophilus* hypothetical protein (EMF46290) (38% in 79 aa) (2e-12) |
| **pGIAK1_19** | 15697-15831 | 44 | Hypothetical protein | No hits |
| **pGIAK1_20** | 16264-16527 | 87 | Hypothetical Protein | *Bacillus licheniformis* hypothetical protein (WP_017474126) (60% in 86 aa) (4e-31) |
| **pGIAK1_21** | 17342-16941 | 133 | Hypothetical protein | *Alkalibacillus haloalkaliphilus* hypothetical protein (WP_017187604) (31% in 132 aa) (6e-04) |
| **pGIAK1_22** | 17465-18829 | 454 | Hypothetical protein | *Bacillus* sp. 123MFChir2 hypothetical protein (WP_020060197) (27% in 415 aa) (3e-30) |
| **pGIAK1_23** | 20047-19478 | 189 | Phage integrase (Tyrosine recombinase) | *B. subtilis* subsp. *natto* phage integrase (BAJ76951) (51% in 189 aa) (1e-60)  Contains phage integrase family conserved domain |
| **pGIAK1_24** | 20436-20987 | 183 | Hypothetical protein | *Desulfotomaculum kuznetsovii* DSM 6115 hypothetical protein Desku_0288 (AEG13919) (29% in 183 aa) (1e-14)  Contains DUF2703 conserved domain |
| **pGIAK1_25** | 21054-21368 | 104 | ArsR family transcriptional regulator | *B. clausii* KSM-K16 ArsR family transcriptional regulator (BAD63371) (72% in 94 aa) (2e-44)  Contains HTH_ARSR conserved domain |
| **pGIAK1_26** | 21422-21826 | 134 | ArsK, glyoxalase family protein | *B. clausii* KSM-K16 lactoylglutathione lyase (BAD63373) (69 in 123 aa) (1e-59)  Contains Glo_EDI_BRP_like_20 domain |
| **pGIAK1_27** | 21941-22288 | 114 | ArsR family transcriptional regulator | *B. clausii* KSM-K16 ArsR family transcriptional regulator (BAD63374) (67% in 109 aa) (3e-47)  Contains HTH_ARSR conserved domain |
| **pGIAK1_28** | 22305-23603 | 432 | ArsB, arsenical pump membrane protein | *Bacillus alcalophilus* ATCC 27647 arsenical pump membrane protein (EJS98284) (83% in 431 aa) (< 1e-100)  Contains ArsB_permease domain |
| **pGIAK1_29** | 23670-24029 | 119 | ArsC, arsenate reductase | *B. clausii* KSM-K16 arsenate reductase (BAD63376) (78% in 116 aa) (3e-63)  Contains LMWPc conserved domain |
| **pGIAK1_30** | 25075-24359 | 238 | CcdA, cytochrome C biogenesis transmembrane region family protein | *Bacillus* sp. L1 cytochrome C biogenesis protein DsbD (WP_017728667) (58% in 237 aa) (5e-99)  Contains CcdA conserved domain |
| **pGIAK1_31** | 25667-25110 | 185 | ResA, thiol-disulfide  oxidoreductase | *Salsuginibacillus kocurii* hypothetical protein (WP_018921583) (59% in 177 aa) (6e-74)Contains TlpA-like family conserved domain |
| **pGIAK1_32** | 25907-26275 | 122 | CadC, cadmium efflux system accessory protein | *Desmospora* sp. 8437 cadmium efflux system accessory protein (64% in 115 aa) (3e-49)  Contains HTH_ARSR conserved domain |
| **pGIAK1_33** | 26279-28453 | 724 | CadA, cadmium efflux P-type ATPase | *A. haloalkaliphilus* cadmium transporter (WP_017187523) (92% in 725 aa) (< 1e-100)  Contains ATPase_P-type conserved domain |
| **pGIAK1_34** | 28542-29153 | 203 | Cadmium resistance transporter, CadD family | *A. haloalkaliphilus* cadmium resistance protein CadD (WP_017187524) (96% in 203 aa) (1e-124)  Contains Cad conserved domain |
| **pGIAK1_35** | 29186-30208 | 340 | Trk family potassium transport protein | *B. clausii* KSM-K16 Trk family potassium transport system protein (BAD62813) (52% in 341 aa) (1e-124)  Contains Pyr_redox_3 conserved domain |
| **pGIAK1_36** | 30240-30629 | 129 | Putative lipoprotein | *Bacillus* sp. 2_A_57_CT2 hypothetical protein HMPREF1013_04389 (EFV75387) (42% in 128 aa) (3e-21)  Contains YtkA conserved domain |
| **pGIAK1_37** | 30655-31245 | 196 | Probably cytochrome C oxidase Cu (A) center assembly protein | *B. halodurans* C-125 hypothetical protein BH2904 (BAB06623) (47% in 197 aa) (4e-53)  Contains SCO1/SenC conserved domain |
| **pGIAK1_38** | 31739-32083 | 114 | ArsR, bacterial transcriptional regulator family | *Staphylococcus hominis* C80 transcriptional regulator ArsR family (EFS20408) (73% in 110 aa) (2e-52)  Contains HTH_ARSR conserved domain |
| **pGIAK1_39** | 32258-32872 | 204 | CadD, cadmium resistance transporter | *B. cellulosilyticus* DSM 2522 cadmium resistance transporter, CadD family (ADU31328) (73% in 202 aa) (1e-89)  Contains Cad conserved domain |
| **pGIAK1_40** | 33028-33831 | 267 | Replication-associated RepB  Chromosome partitioning ATPase | *B. cereus* VD196 chromosome partitioning protein IKG_05572 (EOO61316) (49% in 259 aa) (3e-84)  Contains ParA conserved domain |
| **pGIAK1_41** | 33815-34066 | 83 | Hypothetical protein | *Roseibium* sp. TrichSKD4 exodeoxyribonuclease VII large subunit (EFO34577) (35% in 86 aa) (1.8)  Contains PHA03246 conserved domain |
| **pGIAK1_42** | 34658-34461 | 65 | Hypothetical protein | No hits |
| **pGIAK1_43** | 34846-35379 | 177 | DnaJ domain protein | *Bacillus thuringiensis* serovar *sotto* str. T04001 molecular chaperone DnaJ (EEM38630) (56% in 146 aa) (3e-47)  Contains DnaJ conserved domain |
| **pGIAK1_44** | 35426-35698 | 90 | Hypothetical protein | No hits |
| **pGIAK1_45** | 35715-36152 | 145 | Hypothetical protein | *B. cereus* AH187 hypothetical protein BCAH187_E0024 (ACJ82920) (37 in 133 aa) (1e-12) |
| **pGIAK1_46** | 36201-36512 | 103 | Hypothetical protein | No hits |
| **pGIAK1_47** | 36664-36876 | 70 | Hypothetical protein | *Virgibacillus halodenitrificans* hypothetical protein (WP_019377654) (31% in 68 aa) (0.73) |
| **pGIAK1_48** | 36901-37056 | 51 | Hypothetical protein | No hits |
| **pGIAK1_49** | 37723-37848 | 41 | Hypothetical protein | No hits |

^*^ GenBank non-redundant (nr) protein sequences database; the minimal length of overlap is 80 %.
